# Walking the Talk: LGBTQ Allies in Australian Secondary Schools

**DOI:** 10.3389/fsoc.2021.611001

**Published:** 2021-07-27

**Authors:** Mark Vicars, Samara Van Toledo

**Affiliations:** College of Arts & Education, Victoria University, Melbourne, VIC, Australia

**Keywords:** secondary school, Australia, school culture, LGBT, ally

## Abstract

Sexual culture(s) are an active presence in the shaping of school relations, and LGBTQ issues have long been recognized as a dangerous form of knowledge in school settings. Queer issues in educational domains quickly attract surveillance and have historically often been aggressively prosecuted and silence enforced. This paper examines the intersections of straight allies in promoting an LGBTQ visibility and agency in Australian secondary schools. Drawing on interviews with “straight”-identified secondary students, a narrative methodology was utilized to explore the presence of student allies for making safe schools. Drawing on straight secondary students' responses to LGBTQ issues in their schools, firsthand accounts of intervening in heteronorming school cultures focus on experiences of being an ally to address LGBTQ inclusivity in Australian secondary schools.

## Introduction

The problematic nature of social and academic participation in school communities for lesbian, gay, bisexual, transgender, queer (LGBTQ) youth often involves how stigmatizing relational dynamics become the dominant narrative during the compulsory years of schooling Callingham, 2018. However, as attitudes and expressions toward (homo)sexuality are being increasingly influenced by and connected to wider discourses happening beyond the school gates, the increased representation of sexual identities in the public domain suggests the significance of popular culture in peer cultures for providing teachable moments about LGBTQ lives. Dyer (1992, p. 161) notes how, “It is within culture that homosexual identities are formed,” and as sexual diversity is increasingly made visible in mainstream popular culture, the presence of LGBTQ identity, it could be argued, is increasingly queering the quotidian. The diaspora of queer characters flowing off the screen into living rooms is, as Eng (2003, p. 4) notes, “providing new ways of contesting traditional…kinship structures of reorganizing…communities based on the assumption of a common set of social practices.” The emergence of the “new” pro-gay as a performative construction (Gorman-Murray, 2013, p. 222) is illustrated in the recent Canadian TV sitcom *Schitt's Creek*, which received critical acclaim and community praise for its representation of inclusion. Sexual diversity is introduced with the initially pansexual/gay character of David Rose, who as he goes about his everyday business, represents the normalization of homosexuality in a community that articulates new discursive configurations of embodying a pro-gay straight identity. This re-textualization, we suggest, not only queers the disciplining spaces of everyday life, but could be read an illustration of “acts of activism…within ‘everyday' places” (Hickey-Moody and Haworth, 2009, p. 80).

The notion of re-/textualization as a pedagogical context started us thinking about how gay–straight alliances and associations among youth in schools offer up “spaces of possibility for new kinds of action, new kinds of learning, and newly emergent subjectivities” (Mayo, 2017, p. 1). We started to wonder about how being an LGBTQ ally could be read as “acts of activism [for interrupting homophobia]…within ‘everyday' places” and what that could mean within Australian school communities (Vibert and Shields, 2003; Jones and Hillier, 2012). Does the presence of LGBTQ allies in schools interrupt narratives of the sexual “norm” and create the space to be different?

In Kjaran's (2017) study of heteronormativity in Icelandic high schools, the reproduction of the sexual norm was explained by a gay participant in terms of how

The kids at school talk very openly about their sex life [of heterosexual students] and of others and it was expected that I did the same. I couldn't do this, I couldn't participate in this kind of discussion, and I felt therefore somehow different, like I was less valued as a man (Kjaran, 2017, p. 99).

Being is, as Barker (1989) notes, a transforming relation and is often “a mode of response to the very forms of power that each day reproduces it” (p. 88). Being is invariably a dialogic experience, and in this paper, our thinking about being an LGBT ally was framed by the effects of intra-action for re-textualizing the everyday spaces of schools. Our aim in this paper is to consider the interruption of microflows of heterosexism and homophobia by “straight”-identified LGBTQ allies in Australian secondary schools, and guiding our enquiry was our interest in hearing insider experiential accounts of affirming LGBTQ sexuality in Australian high schools. There are few documented narratives of straight-identified allies, and as narrative researchers, we are naturally drawn to stories about people and their place in the world. It has been discussed that narratives about people and their lives act “as both a means for knowing and a way of telling about the social world” (Bochner, 2001, p. 155), and we purposefully decided to keep the investigation broad. In doing so, we invited the participants to reflect on and retell their lived experiences of encountering and resisting heteronormative and homophobic expressions in their school communities. We were cognizant that the stories we were going to be told would not purely be concerned with the self and considered the participant narratives as not only biographical accounts of life events, but also as interpretations that communicated “a way of understanding and analyzing, the involvement of self with others within their combined discourses” (Chang, 2008).

In starting to think about the axiomatic, regulatory norms of the pedagogy of social and cultural practices in educational domains, we acknowledge how heterosexism and homophobia are all too often tacitly institutionalized at the macro level in Australian schools (Cumming-Potvin and Martino, 2018) and, as a narrativizing practice, the importance of addressing the wider sociocultural conditions in which this study is located are now addressed.

## It Ain't no Mardi Gras Here: a (Brief) Australian Context

It is difficult to provide a “grand” narrative of homosexuality in Australia due to the geographical and legislative differences that characterize federation. It is a notable feature of the Australian context how changes in legislation have followed a change in public attitudes toward homosexuality, but these attitudes have varied widely from state to state. Contextualizing the legislative variances to which LGBTQ individuals have been subject makes it possible to understand the checkered history of homosexual law reform and experience in Australia. In 1973, Australia decriminalized consensual homosexual acts that took place in private domains; however, it took 22 years for all Australian states to enact the repeal with Tasmania being the last state to remove sexual acts between consenting adults as a criminal offense in 1997 (Power, 2011; Willet, 2013). Positive attitudinal shifts across Australia toward homosexuals did not advance uniformly or were widespread, and a further reinforcement of the Australian government's staunch antihomosexual stance was made easier in the 1980s due to the pervading presence of the HIV virus, which had started to permeate a moral panic narrative within Australian society. Gay men, specifically, during this decade were universally represented by the media as “AIDS carriers” responsible for the infection of “innocent” heterosexual people through their reckless sexual activity (Lupton, 1999, p. 51). This moral panic reflected public sentiment and was echoed in the rise of vigilante antigay groups, who took to the streets and “gay bashing” as a form of retribution (Robinson et al., 2014; Schenkel, 2017). Moral panic and fear-mongering by religious groups and conservative sectors of the media called for a return to Victorian-era values and strict adherence to biblical notions of chastity and fidelity.

In 1993, the gay panic defense was called into question in Mudgee, New South Wales, when Malcolm Green murdered Donald Gillies because he alleged that he entered his bedroom naked and made sexual advances toward him. In the ensuing legal battle Green was found guilty of manslaughter, a reduced conviction, due to what his defense team argued as reasonable provocation. The Honorable Justice Kirby, an openly gay man in the judiciary, was only one of three sitting judges to dispute the finding and warned of the implications of such a decision. He remarked that, in a heterosexual case of a similar nature, such an excuse for violence would be unacceptable.

Legislative battles around the issue of homosexuality emerged once again in the Australian public domain in 2004 with the battle for the legalization of gay marriage. Debated by a predominantly heterosexual Australia, the argument garnered divisive media commentary. The then Prime Minister of Australia, John Howard, declared that the institution of marriage was a sanctity that could only take place between a man and a woman. The idea of legally recognized same-sex relationships for conservative heterosexuals, according to Edwards (2007), attacked the core of idealized notions of masculinity and patriarchy and created fear that both could become redundant. In 2017, 61.6% of the Australian population voted affirmatively for gay marriage; however, the debate that preceded the ruling was unrelenting and damaging with many LGBTQ individuals reporting “stigma-related stress” as a result of homophobic reporting, advertising, and discussion (Ecker et al., 2019, p. 213). Nadal et al. (2010) study the effects on the LGBTQ community during the Australian gay marriage plebiscite and report that interpersonal microaggression, which refers to day-to-day forms of subtle or unconscious discrimination often articulated in language, was heightened during the lead up to the vote (Perales and Todd, 2018). The detrimental effect on LGBTQ people was described by a participant in the Chonody et al. (2020, p. 58) study, who said, “This postal farce has done nothing but erode the Australian people's sense of community and turn what were once friendly neighbors against one another.”

As Australian public attitudes on homosexuality vary from state to state, legislation continues to be produced that has the potential to diminish the lives of LGBTQ people. In 2013, the federal government passed the Sexual Orientation, Gender Identity, and Intersex Status Amendment Act with an exemption that continues to allow religious schools and organizations to discriminate based on sexual orientation. In 2019, in Queensland, the Health Legislation Amendment Bill 2019 (QLD) was passed by a narrow majority. Although this legislation prohibits shock therapy treatments for LGBTQ youth, it does not ban conversion therapy that takes place outside of healthcare domains. Despite small legislative wins, there continues to be policy presented to parliament designed to impede the rights of LGBTQ people. In 2020, Mark Latham, the ex-leader of the Australian Labor party and the current New South Wales leader of the One Nation Party, introduced the Education Legislation Amendment (Parental Rights) Bill 2020 (NSW), which, if instated, will prohibit the teaching of gender fluidity in schools across the state of New South Wales.

The regulation of schools in matters pertaining to sexuality has had a powerful and long-lasting impact and has shaped the formulation and adoption of educational cultures in Australia. Reading the regulation of sexuality from a critically queer perspective can reveal what lies beneath the surface of everyday discourse and leads us to consider how compulsory heterosexuality, a pervading feature of the psyche in Australian culture, is situated in Australian educational domains (Martino and Pallotta-Chiarolli, 2001).

## What Lies Beneath: LGBT in Australian Schools

The promotion of normative sexuality in the Australian educational domains offers a framework of legitimacy that inevitably produces symbolic and material exclusion as evidenced in 1979 with the then Victorian Minister of Education memorandum that was sent to all schools in the state ordering principals not to stock any books or materials that encouraged or promoted homosexuality (Marshall, 2014). Ferfolja (2007, p. 148) writes how in “Australia, Western discourses of childhood prevail, constructing youth as innocent, vulnerable, asexual, unknowing, in need of protection from moral turpitude, and in binary opposition to adults.” Epstein et al. (2002) argue that this notion of “innocence” was developed as a way of maintaining power of authority over and ignorance of sexual behavior and identity, and as young people traverse the corridors of school, the regulation of matters pertaining to sexuality becomes subject to the panoptic gaze and interpellations of moral entrepreneurs. Surveillance and regulation have meant that the expression of sexuality when conjoined with young people continues to be considered as a dangerous form of knowledge in school settings and to be the target of political, social, educational, and legal regulations (Epstein and Johnson, 1998). Although it should be noted that, despite changing attitudes in society at large, little headway has been made in producing more positive and engaging educational experiences for LGBTQ students in Australian school domains. Even though the study of sexuality in Australian educational domains has a relatively recent history (Rasmussen, 2004, 2006; Martino and Pallotta-Chiarolli, 2005; Mitchell et al., 2011; Lea et al., 2014; Ullman, 2015; Grant et al., 2021), educational research throughout the 1990s and 2000s consistently demonstrates how LGBTQ+ youth in Australian schools were at risk, with suicidal behavior and self-harm tendencies at disproportionate levels to their heterosexual peers (Castro and Sujak, 2014). The Hillier et al. (2010) study of LGBTQ+ youth in Australian schools demonstrates and documents the urgent need for an inclusive and focused curriculum to support the needs of LGBTQ+ students and their friends, and research continues to indicate the incidence of negative experiences for Australian LGBTQ+ students (Loutzenheiser, 2015).

Although a comprehensive critical examination of the power and effect of heteronormativity in Australian schools remains unaddressed, counternarratives are emerging (Marshall, 2011; Ullman, 2015; Jones and Hillier, 2016; Ward, 2017; Jones, 2020). However, when steps are taken toward a progressive and inclusive approach to teaching and learning, they are usually short-lived, and in 2010, in the Australian state of Victoria, the Safe Schools Coalition was formed and implemented Australia-wide in 2013. The program aimed to provide

Professional development for school staff;
Guidance and support for teachers around specific issues and concerns or for schools to support individual students upon request;
Printed and digital approved resources for teachers that provide the information and tools to respond to bullying and discrimination;
Support to ensure the school's curriculum and practices are inclusive to students who are same sex attracted, gender diverse, or intersex;

Support for schools in reviewing or introducing antibullying or diversity policies to be safer and more inclusive.

(http://www.safeschoolscoalition.org.au/from-a-safe-schools-coalition-australia-ssca-spokesperson-6) (Safe Schools Coalition Australia, 2020).

However, a backlash by Australian conservative groups put pressure on the program's funded viability and contributed to the subsequent withdrawal of it as a national program in 2016.

It is not the aim of this paper to provide a detailed account of the literature that addresses LGBTQ inclusion/exclusion in Australian schools, but to set the scene for what Ferfolja (2007) argues: how heterosexuality is privileged in many aspects of curriculum although non-normative sexualities are tacitly hidden and framed as the “educational other” (Slee, 2013).

At an individual level, the category of educational other comprises students who are disenfranchised and marginalized within schools through social divisions and hierarchies of worth (Gergen and Dixon-Roman, 2014). At a systemic level, the educational other is routinely perpetuated by an education system that inscribes narrow conceptions of what schooling is and does. Othering has a primeval genealogy, which can make belonging a tricky path to navigate. De Beauvoir (2010, p. 26) writes that “no group ever defines itself as One without immediately setting up the Other opposite itself; and Otherness is therefore, bound by power relations where the ‘known and unknown' are set apart and cast as opposites” (Creutz-Kämppi, 2008, p. 297). The reductive action of “othering” habitually involves a linguistic interpellation of difference, and Guralnik and Simeon (2010, p. 407) discuss how interpellation is “the very mechanism through which ideology takes hold of the individual [and is] the authoritative voice of the State [that] recognizes the individual and hails him into social existence” while simultaneously casting out the other. This interpellation of deficit and the development of a pejorative nomenclature functions as a means of division and boundary making, of “them from us.” Rothmann and Simmonds (2015) study of preservice education students demonstrates how the use of linguistic tools objectify LGBTQ identities and maintain the separation between “them” and “us.” Participants in their research consisted of fourth-year education students who were given a fictitious scenario that centered around teaching LGBTQ students despite being religiously opposed to the idea. The participants' collated responses demonstrated that a large proportion used objectifying terms, such as “it,” “things,” “stuff,” “they,” “issue,” and “them,” in relation to LGBTQ+ people, and the lack of intersubjective connection emphasized the focusing on differences rather than the intersections of connectedness (Okolie, 2003).

If, as we suggest, essentialist notions of sexuality and gender are strongly interwoven into the fabric of Australian school life, then these become the structures that support how heteronormativity becomes institutionalized. The processes and culture that keep it in operation are tacitly understood by those who inhabit and reify the “norm in a country in which, it could be argued, the derogatory ‘poofter,' is the preferred interpellation of stigma that brings a weighted pressure to conform to and perform cis-gendered heterosexuality” (Dowsett, 2003). Studies that have mapped a social geography of homophobia identify locations within schools where heteronormative practices are most frequently and aggressively prosecuted. Pejorative schoolyard jokes and jibes about “queers” habitually inculcate heteronormative scripts (Ellis and High, 2004; Vicars, 2005, 2008a). Rofes (1995) indicates how words such as “poofter, gay, queer, homo, lezzy” are highly meaningful in that they continue to constitute what is and is not considered “normal” within daily school life and peer culture, and Woodford et al. (2013) notes the causal relationship of hearing heterosexist language and feelings of social isolation

It goes without saying that LGBTQ lives are made up of a finite number of crucial interactions, and the significance of interpersonal relationships within social networks extend an influence on how individuals think about themselves and their peers. Putnam (2004) suggests the connections of individuals in and between groups can be substantially beneficial to members. Cassity and Gow (2006, p. 44) find that the biggest challenge for all secondary students was to “seek out a community to which they could safely belong,” and for LGBTQ students, this can still be a troublesome task. Grant et al. (2021, p. 2) note the “dearth of Australian research exploring the impact of LGBTQ student groups on school cultures,” and this may well be to the emergent nature of such groups in Australia. To the best of our knowledge at the time of writing, there is only one gay–straight alliance reported in operation in Australia, and it started in 2016 at a Victorian Grammar school.

## ALLYSHIP: What Is it? What Does it Mean?

Schooling can often be a rite-of-passage milestone for LGBTQ students in which they get read as dislocations in the normative discourses of practice (Robinson et al., 2014). For many of us, the experience of being the gay kid, the gay student, the gay sibling, the gay friend is drawn from the vagaries of our encounters and interactions and is shaped by the verities of existence. We have written elsewhere how the most powerful parts always go to heterosexual protagonists for reinforcing or resisting the heteronormative script in everyday life:

*Throughout my childhood and into my adolescence, I became accustomed to not making sense. Having a growing awareness of not being straight enough, involved me rethinking “I” in relation to the wider communities in which I sought belonging. I am conscious how I am/was often perceived as being “too gay” which invariably means being “too queeny,” “too flamboyant,” “too visible”* (Vicars, 2012, p. 55).

*I began to realize that my brother was not like the other boys that I had encountered as soon as I entered the institution of school. My brother refused to accept the binary social enforcement of gender. He expressed himself in a socially taboo manner rejecting shades of color and clothing which we had been taught from a young age were suitable for boys. He learned ballet, enjoyed the theater of “dressing up” and refused to play the “holy” sport of football. He was subjected to taunts, physical harassment and societal disapproval. My brother was a minority residing in a town which displayed disapproval and in many cases overt loathing toward his determination and inclination to self-express and be gender fluid. As his sibling, I also became victim to this treatment* (Van Toledo, 2018, p. 113).

Although Tillmann-Healy (2001) research into gay and straight friendships and Gorman-Murray's (2013) study on gay–straight friendships demonstrate the power of proximity and situatedness for enacting attitudinal change and advocating for LGBTQ issues, it would, we suggest, also have an effect on how allies position themselves within their existing social networks (Smith, 2015). Being visible as an ally, it seems, is not only an act of world re-/making, but also of self re-/making. The participants in their stories speak of how their adoption of an ally role was constructed through daily school experiences and involved a conscious taking-up of a positionality/identity in specific moments and in encounters with others (Grzanka et al., 2015). A couple of the participants spoke of how often this could be a balancing act between providing support, interrupting stigmatizing behaviors, and resisting the suspicion of being queer themselves. These “complex associations” of being an ally of which Mayo (2017) speaks have been suggested to be in the negotiation of straight privilege and queer exclusion in educational domains. The risks that come with speaking out affirmatively for LGBTQ peers is, as Mayo (2017, p. 121) notes, appearing in “contexts or are known to publics in ways they cannot control,” and perhaps the most telling difference between being an ally for LGBTQ peers and formal gay–straight alliances is in the formal and institutionalized structure that supports and scaffolds the latter.

Studies that examine the impacts of gay–straight alliances attest to the positive impacts on academic performance and social well-being their presence has in schools (Kosciw et al., 2010; Walls et al., 2010; Toomey et al., 2011; Smith et al., 2014; Poteat et al., 2015; Baams et al., 2020; Lessard et al., 2020a,b), and although there are resource materials on how to be an ally, there is a paucity of scholarship on the experience of being a straight ally. Grzanka et al. (2015), in examining the concept and identities of straight LGBTQ activism, sought to understand how “a straight ally identity is produced in the social worlds of those who identify as allies and how they came to this identity by way of interactions” (p. 166). The study notes the misleading conflation of being a straight ally with LGBTQ activism and advances a consideration of how straight allyship “represented a form of identity choreography, that was both deeply affective and intricately intentional” (p. 177). Coining the term “identity choreography as way to think through (1) how individuals integrate meanings and knowledge from otherwise discreet social orders, (2) how those meanings are anchored in personal, self-reflexive narratives about identity,” (p. 177) the study notes that the category of ally “should not be rendered monolithic or singular in either form or content” (p. 179) but is contoured as Valentine (2000, p. 257) notes,

“within the context of peer group culture highly embodied and [for young people] predicated on adult notions of heterosexualized gender identities.”

As each of the participants' told their experiential stories of what often lies beneath and beyond the macro surface of school and is seldom immediately visible to the teacher or educational researcher, the instrumental complexities of taking up of an ally positionality was routinely located in the participants' relational interactions with others.

## Methodology

Four participants, three females aged between 15 and 21 and one male aged 16, were snowball sample recruited from within a friendship group and a colleague's young adult family member. The defining characteristics of the sample were the participants' pro-gay views, concerns that negative attitudes toward LGBTQ students were becoming further entrenched in their schools, and how school operations and processes did not meet the needs of LGBTQ students. All of the participants had LGBTQ-identified friends and were already invested in being an ally. They all shared a set of ideological beliefs and values that stand against the heteronormative cultures in school. The participants all came from middle-class backgrounds and had attended or were attending independent selective high schools/selective state grammar schools. They were invited to participate in a 20-min recorded Viber interview, and the purpose of the interview was to identify themes connected to the participants' perceptions and views of what it meant for them to publicly identify and be a LGBTQ ally. The participants were not asked to respond to an explicit research question but were invited to reflect on their school experiences connected to LGBTQ-identifying students. At the start of the interview, the participants were invited to tell their stories from their own perspective. They were informed that the interview would be recorded and edited into a grammatically correct version and represented verbatim.

The small-scale sample of participants was a deliberate part of the study's design, which had the aim to convey rich, thick, detailed “ethnographic miniatures” of lived experiences (Geertz, 1973, p. 318). The interviews drew upon the biographic-narrative interpretive method approach (Wengraf, 2001) to draw out narratives from a participant's own cultural and emotional perspectives. We purposefully chose to move away from the structured interviewing format due to how questions that are devised by the researcher often come from assumptions about what participant's lives might be like (Jones, 2003). We were aiming to gain insight into the unique experiences and positionality of participant's lives in their own words and from their own perspectives (Jones, 2003; Bochner, 2012), and as St. Pierre (2021, p. 6) suggests, “The concept data collection is itself problematic because it points to an ontology that assumes data are separate from human being and so can be ‘collected.”'

Working from an “ethics of care” (Glen, 2000), we explained to the participants the interpretative naturalistic purpose and scope of the interviews as a context for them to speak freely. To ensure anonymity of the participants and their school, any identifying details were changed and pseudonyms utilized. As with any research interview, consideration of power dynamics and consent are an abiding issue but are especially important when conducting research with young people (France, 2004; Mishna et al., 2004; Morrow, 2008). We were mindful of the power dynamics in play that are created by the binaries of adult/child, researcher/participant and that the something at stake in research inquiry (Smith, 2001, p. 5) can often be “the participant.”

Noting the importance of the ethics of representation, Stanley (1993, p. 43–50) suggests how the “'autobiography'.of the sociologist becomes epistemologically crucial no matter what particular research activity we are engaged in” and in declaring our “position” as queerly situated educational researchers, we made it clear to the participants how our personal experience was informing our professional interests (Vicars, 2006). Blackman (2016) notes that researchers often feel that they must maintain an outsider's perspective by not revealing their own personal and emotional connections within research, but we believe such an ontologically situated approach can usefully problematize the research process. Sparkes (1996) and Young (2012) advise that researchers should not be afraid to reveal themselves, their experiences, or investments within their work. Sharing our beliefs and values with the participants, some might argue, presents a problematic bias, but as Sandelowski (1991) notes, the interconnectedness of the stories of participant and researcher rooted in the subjectivities of both works toward building rapport and ease between the interviewer and interviewee and lie at the core of ethical interviewing.

Although Coffey (1999, p. 133) notes, that there remains considerable debate over the degree to which autobiographical “texts should represent the field, the self or both,” we set out with the conviction that a “truth” would be told (Sikes, 2000, 2009, 2010). Stories we suggest are interpretations of the world that require an audience to make its own meaning, and throughout the interviews, the importance of participant and researcher relationality and positionality to the issues affecting LGBTQ students in high school domains became a telling relation.

## Analytical Framework

Sikes (2001) points out how stories told are subject to time, place, and personal involvements, but so too is interpretation, and in analysis, we drew upon the framework of queer theory for thinking about how identity and subjectivity becomes *materialized and inscribed* within social encounters and for understanding how ways of being are made visible within intricate relations with others and are sites of identity formation, self-definition, and affiliation.

In analysis, we utilized the trope of the rhizome from which to understand the significance of interpersonal relationships within social networks for countering LGBTQ microaggressions. We endeavored to understand how the participants subjectivities as allies were negotiated by being “embedded in webs of relationships with others” (Davies, 2015, p. 680). The rhizome that has been suggested is:

unlike a structure, which is defined by a set of points and positions, with binary relations between the points and biunivocal relationships between the positions, the rhizome is made only of lines: lines of segmentarity and stratification as its dimensions (Deleuze and Guattari, 1987, p. 21).

It proved useful for addressing the problems inherent with speaking out of a cultural context and positionality and afforded a means for acknowledging location. The participants in their stories narrated how

meaning emerges not from the thing-in-itself but from its relationships to an infinite…number of things. In this complexity we understand from another angle that there is no….final meaning of anything; meanings are always evolving in light of new relationships… new horizons (Kincheloe, 2011, p. 214).

The rhizome, as St. Pierre (2021, p. 4) notes, is “deliberately anti-method” but useful for “reorientating thought,” and in the following stories, the participants addressed how their ally behaviors were contoured between the problems of individual expression connected to their participation in the wider school community. Their stories are of interpersonal relationships and social networks and in the telling show the gestalt involved in the expression of being an LGBTQ ally.

## Walking the Talk: Doing Sexual Diversity as an Everyday Thing

Sophie, a 21-year-old university student studying a postgraduate degree in arts, had attended both a mixed-gender public high school and a selective high school in predominantly middle-class, affluent suburbs. Reflecting back on the differences in attitudes between the schools, she spoke of how,

*at my local public high school, any thing to do with being gay wasn't discussed, and it was a derogative thing to be called, but I changed schools in year 10 and went to a selective all-girls school, and it was so different—there were these lesbian couples openly dating. There was a group called SSFYF: Same sex attracted youth and friends that was held once a week after school. It was kinda like a support group, and they had a display board in school where they would post stuff. Being at single-sex school was a very different experience; it was much more open and more switched on to LGBT issues. At school assemblies, they talked about being gay and about the importance of being out*.

*Coloring all of this was that, in our school, there were some openly gay teachers, and this made the environment feel more diverse and accepting. There was this one teacher who presented as very masculine. She was quite androgynous, and we loved her as she was quite active in changing the school attitude toward LGBT. The school was very supportive in terms of allowing student gay–straight clubs and societies and publicly talking about LGBTQ issues and getting the message across to everybody in the school. There were a few people that had come out as Trans, and in a single-sex school, that could have been an issue, but it really wasn't. The school population were quite switched on, and it wasn't just LGBTQ issues that were being discussed, there were numerous clubs that were talking about a whole range of issues. There were feminist clubs, climate change clubs—you get the idea? We thought about ourselves as progressive and powerful women, and we spoke of ourselves as being smart women*.

*Attitudes toward LGBTQ were part of a wider ideology that was happening at that school. These were mainly coming from the student body, and even though there were examples of it being okay being gay or trans, at the same time, I and other people were still scared. There are a lot of my friends who are now ‘out' that were not ‘out' at school, and I think that is because, at our other schools—the ones we had been to before—people would openly demean gay people, and discrimination happened. I think people were waiting it out ‘till they could be sure it was safe. Even though it was accepted then, it is not as accepted as it is now. What was happening at the school was good, but I don't think it would have changed people's inherent biases that they had internalized growing-up*.

*People were still unlearning, and in general, the politics of queerness was changing really quickly at that time—public attitudes were changing, and there were things that people would say 10 years ago that are now not acceptable. Programs like* Modern Family *had started when I was in high school, and popular culture was becoming quite queer, and all that stuff on the TV was quite shaping how people at school were behaving and what they were saying. My learning around gender and sexuality happened mainly in friendship groups at school. My friend transitioned during our final year at school. He sent an email around saying he was transitioning and that he was changing his name to Paul and could we please use he/his pronouns and address him as Paul. I was very happy that I and my friends could show support by using his preferred pronoun and you know just being visibly on his side. At the end of year prom, he won the best-dressed guy category at the school ball, which is voted for by the student body and usually someone's random date would win it. This was a real validation for him, and when he won it, everybody just burst into applause and was genuinely happy for him. I didn't really think about showing support for LGBT students, it was something that was not really questioned in my friendship group, it was just something that we did. Why wouldn't we? That would just have been stupid*.

Ruth, a 15-year-old, year 9 high school student at a selective fee-paying Melbourne school in an affluent middle-class suburb of Melbourne initially commented on the “pack mentality” in her school and how it frames what is and is not possible in countering homophobic commentary:

*When 15-year-olds get in packs, they have the most offensive humor possible. The more people realize that the things they say can hurt people, they might actually look and take a step back and think about their actions. When they are in a big group, they get caught up in the whole “Oh, when I say this, people laugh” and “this is what everyone is doing.” Phrases such as “that's so gay” are gotten away with because, when they are called on it, they are like “oh no, it's a joke—I didn't mean it.” A lot of my year group kind of decide not to see the problems that they cause and that are behind what they say and whether that means they are promoting stereotypes. Those comments such as “that's so gay” are really bad if you are gay or struggling with your sexuality or even if you are out. It really does matter, and so do rumors. I have had some friends who have been badly hurt by some antigay stuff that people have said, and you need someone to challenge it. I'm not the only one who does that*.

*There are quite a few people in some of the groups I mix with that take it on as some people they mix with that speak in a derogative way about the LGBTQ community without even realizing it. It's very normalized at school, so I feel like the more you can make that not normal and call it out, then whenever you do that, it might actually get in to their heads that it is not alright. There were a couple of people who were friends of mine at first, and then they made anti-LGBTQ jokes and comments and were watching other friends of mine who are constantly watching how some of the boys in our group who are a bit effeminate are doing and what they were saying. They then make jokes about them. I think being a girl makes it easier, especially in high school, to challenge that as boys are much more pressured to appear cool. I've always had strong opinions, and ever since year 7, I have made my views seen on human rights issues and that kind of thing. I jokingly got called a “feminazi” as everyone in my year group knows what I do. People know not to make those kind of jokes—anti-LGBT jokes around me ‘cos if they, they will antagonize me, and then I will say something back. People know that I will challenge stuff that is offensive, and ‘cos they are aware they don't make those kinds of jokes when I am around. There haven't been any consequences for me speaking out, and there are some people who have said, since you said X, Y, Z, I have actually thought about it, and you had a point. One of my friends is out, and she is not afraid to deal with people if they are being homophobic or sexist. She does get teased but not to her face…not many of my year group would directly say anything. I don't know if that makes it better or worse as the name calling is all very much behind people's backs. I have never been bullied for speaking out. Joe, one of my friends, does a really good job of closing negative stuff down, and he does it in a quiet way. He is in a couple of friendship groups, one of which is the jock/sporty group, and there are three individuals that are seen to lead the charge on saying anti-LGBTQ stuff. Joe says things like “Hey, that's not okay,” or he redirects the conversation. He always takes it on. I am in a friendship group with three gay kids, and they are happy and positive about school. They are quieter than my other friends, and they are not in the big circles of kids that hang around the school. They find the big groups uncomfortable and they say if they are ever stuck in a situation with a lot of kids they don't know or with the jocks that are known to make snide comments, they feel nervous*.

*I don't think the teachers are good at dealing with comments that are made and that they hear. It has got to the point where those kids who say stuff about gay people don't care because the teachers just give them a slap on the wrist. Whilst the teachers don't tend to make it seem like its okay, they might not really know what to do about it, and they pass it off as if those people are not laughing about sexuality but something else. They (the teachers) want to look like they are supporting us, but really it's not anything more than them saying, “Don't say that's gay, we're all part of the school.” Unfortunately, 15-year-olds don't exactly listen to that. At our campus, the year 9 is split off from the rest of the school, and the main school has got really good stuff going on, but it is mainly student organized, but teachers are supportive of it. There are LGBT posters, displays, and on a couple of teacher's offices, there are rainbow flags and helpline flyers for LGBT youth. Support on the main campus is made clear. With social media, Pride month was everywhere at school, and a lot of kids know June is Pride month. In my age group, everyone knows the rainbow flag is the LGBTQ symbol. During Pride months, we (me and some of my friends) got rainbow badges that we pinned on to our blazers, and I've still got mine on. People I know came up to me and said, “People will think you are gay with that on your blazer,” and I responded “Is that really such a bad thing?” During Pride month, me and my friends made a speech at the Year 9 assembly as we realized we hadn't done anything asa year group to acknowledge Pride month. We wanted to tell our year group what it is about and why people should do something for it. I talked a lot about making sure schools are the most comfortable environment given that a lot of us could be questioning who we like and who we are, and a negative comment could be detrimental to that. Some people came up to me after the assembly and said they really liked what I had said. The head of year 9 came up to me and said that what we had done made her emotional, and quite a few of the teachers congratulated me for organizing it. Our presentation was positively received, and there was no gatekeeping by the school. At the main campus, they even put rainbow flags, and they also had a speech at their assembly. My year 10, 11, 12 friends have started an LGBTQ youth support group, and we get together to talk about how the school could be more supportive. In the older year groups, there is a great community and a lot more kids are “out.” The year 10–12 teachers are starting to talk with the kids that identify as LGBT1 and are asking them what they can do to help. They are saying things like “We want to learn from you guys.” “What could we do to help more?” My friend Sage has started to transition, and everyone in my friendship group uses her new name and makes a real effort to acknowledge they are gender neutral. Sage's transition has, on the whole, been positively received, and no one has questioned her pronouns or anything like that, but I think that is more due to trans ignorance*.

Joe, a 15-year-old male friend of Ruth's who attended the same school and was in the same year and friendship group, articulated an experience of how being a male ally is made more problematic.

*We have a couple of kids that have come out this year, and so far, the response has been Okay. Nobody gives them direct flack, but behind the scenes, there is a lot of homophobic remarks made in my sporty friendship group, stuff like “He's a faggot.” They say it when they won't get caught as nobody wants to be in the direct line of fire, they are too scared of being homophobic to those kids' faces as they afraid to cop the consequences. I have two friendship groups, the sporty group in which there is a lot of homophobic behavior and the friendship group, which Ruth is part of. Ruth has kind of rubbed off on all of us, and we have learnt from her that there is nothing wrong with being gay. In the sporty group, it is harder to challenge as they tend to give shit to people who stand up for gay people, and they accuse them of being gay too. If you do say something back, it can get quite dark, so the choice is don't say anything and go along with it or get yourself subjected to verbal slurs. Ruth's group is totally different, we all have similar beliefs, and it is known around school that it is not cool to say antigay stuff. That group has a vibe around school, and we are tight*.

*Schools could improve by putting the word out more to stop homophobic language as that is so normalized, and I hear it so often, and it is hard to question it with some people. I feel if teachers took more of stance, I could have more of an impact by picking people up on what they say, and I should. I would shut people up if I knew someone was gay and they were getting shit in a group. I have a responsibility*.

Nina, a 20-year-old University student who attended a public high school in 2017 in an affluent middle-class suburb of Melbourne commented,

*I get really offended by homophobia. I don't understand it—I really don't understand it! There are lots of LGBTQ kids at my school, which is in a progressive suburb, and I think there is a socioeconomic difference. I don't know how to say this, but less academic kids tend to use more pejorative language, and I think being gay is more of an issue for them*.

*My school was very accepting, and it celebrated Pride day, which was a whole school initiative that tried to make us more literate about LGBTQ issues. The school wanted LGBTQ students to feel welcome, and posters were put up around the school, and there was a special assembly, but it never got introduced in the curriculum, so it never became a learning objective, so it felt a bit like the checking of boxes*.

*My friends were/are gay, and my best friend was out at school in year 11/12 and is non-cis. Our friendship group put a lot of effort in to updating the wider school community on the use of pronouns after they told me they wanted to be addressed as “they/their.” We started a campaign to get the school to put in gender-neutral toilets, but they refused, which was so bad. I am cis presenting, and there is nothing obvious about me that others can latch onto, so I have had a smooth ride ‘cos I fly under the radar even though I now identify as bisexual. I don't see coming out as being all that useful even no, as being queer can get you othered and puts you into other people's boxes*.

## *Discussion—*Straight but Not Regular

The participant stories show how the taking-up of an ally positionality was representative of a mindset that had much to do with their negotiation of their identities as young adults. Advocating for LGBTQ+ issues and being an ally appears to be grounded in and affected by the participants' relational interactions with others. Articulating a pro-gay sensibility was, for all of the participants, grounded in a context of a commitment to social justice issues and embodied speaking back a truth to power. Such a stance bears the features of what Foucault (2001) calls parrhesia, an act of speech that is characterized by a commitment to speak freely with openness and honesty and with criticality and that has the capacity to cause offense and be a risky endeavor. Parrhesia as a knowing and telling relation to being in the world speaks of a deep personal engagement, interaction, and investment with the “what” and “how” of the material-discursive is put to work.

Ruth's comment on the pack mentality in schools echoes Joe's understanding of the personal investment required in countering homophobic commentary. She explained how adopting an ally stance to refute homophobic discourse required taking up a position in which it was impossible to maintain a psychic distance and detachment from name-calling. Ruth, in her narrative, spoke of herself as a summoned subject: “the self constituted and defined by its position as respondent” (Ricoeur, 1995, p. 262), which is echoed across the participant's stories. Ruth explained how adopting an ally stance required actively taking up a position in which it was impossible to maintain a psychic distance and detachment from name-calling and often involved overcoming of personal discomfort and could incur social consequences.

The affective aspect of belonging in and to friendship groups and how gender determined how being an ally is made easier or more difficult is illustrated in Joe's reflection. Joe, a young straight male, straddled two social groups and spoke of, in the wider school population, how his countering of homophobic commentary in the dominant sporty group meant he had to be a “quiet ally” for fear of the repercussions. Athanases and Comar (2008, p. 13) note how

Much of the bullying nature of name-calling is tied to power, position, and peer pressure. Language is pragmatic, purposeful, and meaningful—and deeply related to social interactions, to relationship formation…Name-calling among youth, for example, establishes in-group affiliations and is a form of bullying and aggression often intended to establish a public identity. Boys exhibit more overt direct bullying behaviors than girls [and] Policing gender norms is key, especially in adolescent males' homophobic speech that tends to target other males.

Joe's understanding of being an ally was connected to his understanding of the personal consequences of transgressing hegemonic social and cultural roles aligned with normative expressions of gendered sexuality. In all male sport-orientated social groups, Joe had to negotiate separation in the ways that he belonged, acted, spoke out, and represented himself as a straight male and an ally. His narrative reflection on the complexity of his lived experiences makes material the deconstruction of the self as subject caught between the binaries of gay/straight, belonging/not belonging and exposes the contingencies of identity and subjectivity (Warner, 1999). To move beyond these binaries is always problematic, and his vigilant attendance to how the self is made problematic was framed by what it is possible to be and do within the governing structures of peer groups. Ruth commented on how *being a girl makes it easier especially in high school to challenge that as boys are much more pressured to appear cool* and as Seidman (1993, p. 130) note,

“The logic of identity is a logic of boundary defining…The social productivity of identity is purchased at the price of logic of a hierarchy normalization and exclusion.”

The importance and centrality of gender as a governing pedagogy in the participants' stories situate it as a key factor in how belonging is constituted in peer groups and how it determines the ease of taking up of an ally position. It is interesting to note how Sophie's experience in a single-sex school provided a very different narrative and indicates avenues for future exploration.

Nina raised how socioeconomic differences framed attitudes toward LGBTQ issues in schools and suggested how, within her mainly middle-class “progressive” suburb, LGBTQ was positioned by the school as being part of a social justice agenda. All of the participants had attended or were attending fee-paying selective schools in affluent middle-class suburbs, and the context of class and parental attitudes on the reproduction of and resistance to homophobia has to be considered as an attributing factor.

Literature that has examined sociocultural attitudes toward sexual minorities (Adamczyk, 2017; Powell et al., 2017) asks whether family socialization is instrumental to the “forming and norming” of attitudes or if educational attainment was significant. People from more progressive backgrounds, it is suggested, can be more likely to affirm a sexual minority identity (Schnabel, 2018), and such an assertion suggests that the formation of attitudes intersects with other emblematic indicators. The situated sociocultural capital of the participants' home environments was progressive thinking, affluent middle class in which the average level of parental education was tertiary. If the formation of pro-gay attitudes is situated within social, cultural, and geographical locations, then as (Valentine, 2007), notes attitudes toward exclusion/inclusion are informed by and within the intersections of people's lives and identities. Vicars (2014) understanding of how context, temporality, and positioning can determine adolescent experiences points toward the instrumental role that families play in the development and enactment of doing gender and sexuality in childhood. Brandt (2001, p. 12) suggests “how that which is felt internally as ‘personal experience' is intimately connected to the institutions outside the self that foster and promote such feelings, and if the particularities of parenting became a telling relation(ship), then the ‘family' as a site of a discursive set of social practices inevitably has to account for the broader social and cultural dynamics in which childhood and adolescence gets done” Britzman (1997, p. 194). Invested in the discursive reproduction of the traditional Western family is the making material of discourses that discipline or not (homo)sexuality as a deficit or risky enterprise. Surtees and Gunn (2010, p. 42) note how “Families routinely inscribe normative identity work and further research is needed into how the habits of hearth and home can be an influencing factor in how social inequalities and injustices are negotiated in family practices” (Taylor, 2012; Chan and Erby, 2018).

Although the influence of individual school profile, suburb, socioeconomic status, and parental educational attainment cannot be discounted as underlying factors in the formation of how the participants came to embody an ally identity, they all indicate how being an ally involved the institutional structures and teacher's professional practices. Nina noted that LGBTQ needs to be embedded in school culture and in the teaching and learning curriculum content. She suggested how school engagement with LGBTQ could have been done better and that the commitment to creating inclusive practices in schools was mainly occurring in and emerging out of student-led efforts. Joe reinforced the importance of senior leadership critically engaging with sexuality related diversity to promote inclusive environments and connected how the macro structures of school influence what he feels he could achieve as an ally. Ruth remarked on the importance of effective leadership on LGBTQ issues by teachers and how teachers should be seen to address exclusionary practices and create an environment and school culture that ensures that discrimination on the grounds of sexual orientation is unacceptable and is challenged. Sophie noted how a lesbian visibility among staff set a tone for making sexuality equality visible that maximized awareness of the presence of gender and sexual diversity in the school and was instrumental in changing perceptions, hearts, and minds. These perceptions echo much of the existing research into the importance of effective leadership on LGBTQ issues in educational domains (Griffin and Ouellett, 2002; Barnet et al., 2006; Vicars, 2008b; Jean-Marie et al., 2009; Branch et al., 2013; Boyland et al., 2016; Lee, 2020) and highlights the importance of school leaders/teachers making visible that which often lies beneath and beyond the surface of the everyday business of school. Articulating a counternarrative that interrupts the silencing is connected to teachers overcoming personal discomfort, and the Ezer et al. (2019) study of Australian teachers reports how their participants often expressed feeling confused and hesitant in dealing with the negative impact of heteronormativity on LGBTQ students. Payne and Smith (2013, p. 2) note how

…individuals' behavior or attitudes create a “negative” school climate where student safety and belonging are threatened. Understanding schools in this way does not account for institutional heteronormativity, which is a fundamental organizational structure through which schools function and the people who occupy school spaces interact with one another.

Such experiences can be interrupted when same-sex attracted people have a strong community connection (Hanckel and Morris, 2014; Swannell et al., 2016), and such connections can disrupt the discourses and practices of the LGBTQ educational other (Slee, 2013).

In Ruth's case, she took it upon herself to actively educate her year group school community, and affirmative visibility has long been recognized as important in countering stigma and providing inclusive experiences for LGBTQ youth in schools. Bird and Akerman (2005, p. 24) noted 15 years back how

“educational and social interventions aimed at addressing social exclusion may lead to changes in individual self-concept, increased well-being and more developed social networks.”

From the participants' stories, there has been some movement in the macro interruptions to the everyday discourses of normalcy in their secondary schools, and this appears to be connected to the change in discourse beyond the school gates. Sophie referenced the role that popular cultural representations played in her friendship group in reference to the wider epistemic shifts informing attitudinal changes toward LGBTQ. Ruth described how she received teacher support for delivering a year group assembly to mark Pride month and how the visible display of LGBT posters and rainbow flags signaled on campus LGBTQ support. As an index of the changing material reality of LGBTQ+ visibility Ruth referenced how drawing on LGBTQ artifacts from the wider community was an important part in maximizing awareness of attitudes toward gender and sexual diversity in schools. Nina spoke of how Pride day became a whole school initiative, and the presence of artifacts and celebration of Pride and LGBT history month the participants expressed were significant for assisting them to interrupt performatives of hetero-normalcy and to “provoke a critical social realization” in the wider school community (Alexander, 2005, p. 411–412).

## Concluding Comments

To fully understand the complexities of straight-identified youth taking up an ally position in secondary school communities requires more research. The participants' stories show the biggest challenge in enacting allyship and pro-gay transformations in their schools was as Cassity and Gow (2006, p. 44) note, to “seek out a community to which they could safely belong.” Belonging and the shaping presence of gender appeared to be central to how they made visible an “ally identity” in their relationships with LGBTQ friends and normalized sexual diversity in the wider school domain. Foucault (1977, p. 176) suggest how, “if sexual Otherness is habitually positioned in relation to what is considered as the norm, and in doing so legitimates the norm as the ideal, normalization becomes one of the greatest instruments of power, the power of normalization [that] imposes homogeneity.” The participants' experiences of negotiating friendship, affirmation, and inclusion are messy and are as Cvetkovich (2003, p. 2) notes “…connected to other histories.” Unearthing a genealogy of allyship is beyond the scope of this paper, but it would fair to say that being an ally has become a significant and defining moment in the lives of the participants. Giroux (1988, p. 292) claimes, “The pedagogical value of resistance lies, in part, in the connections it makes between structure and human agency on the one hand and culture and the process of self formation on the other.” Speaking out about the interface of straight culture on queer sexuality, Sedwick (1994, p. 2) notes,

“The knowledge is indelible, but not too astonishing, to anyone with a reason to be attuned to the profligate way this culture has of denying and despoiling queer energies and lives…Everyone who survived has stories about how it was done.”

Heteronormativity and homophobia maintain an affirming presence in Australia, and in this paper, we have endeavored to tell stories that are not usually heard and that we suggest are instrumental to making visible LGBTQ discourse in Australian schools. The participants pro-gay relational interactions with others are, we hope, a promise of an emerging change in school communities and an indication it just might be possible to start to think of a time when the presence of LGBTQ+ allies in schools' render sexuality-related diversity and inclusion as “fairdinkum” and as “Aussie” as Vegemite.

## Data Availability Statement

The raw data supporting the conclusions of this article will be made available by the authors, without undue reservation.

## Ethics Statement

The studies involving human participants were reviewed and approved by Human Research Ethics Committee, Victoria University. Written informed consent to participate in this study was provided by the participants' legal guardian/next of kin. Written informed consent was obtained from the individual(s) for the publication of any potentially identifiable images or data included in this article.

## Author's Note

The contribution this article makes to the academy is in the reflection on how young adults position themselves as allies in Australian secondary schools to interrupt heteronormative discourses. The paper suggests how such positioning is contoured by and in social friendship groups, is gendered, classed and connected to wider social and cultural discourses. Drawing on first hand accounts that describe the experiences of countering stigmatizing and heteronormalcy in school domains, the notion of being an ally is contextualized within the wider Australian landscape of legislative frameworks and attitudinal shifts toward LGBTQ. The article invites the reader to consider the relationship between the macro and the micro and the importance of relationality for resisting homophobia in secondary schools.

## Author Contributions

All authors listed have made a substantial, direct and intellectual contribution to the work, and approved it for publication.

## Conflict of Interest

The authors declare that the research was conducted in the absence of any commercial or financial relationships that could be construed as a potential conflict of interest. The reviewer JG is currently organizing a Research Topic with one author MV.

## Publisher's Note

All claims expressed in this article are solely those of the authors and do not necessarily represent those of their affiliated organizations, or those of the publisher, the editors and the reviewers. Any product that may be evaluated in this article, or claim that may be made by its manufacturer, is not guaranteed or endorsed by the publisher.
